# High Genomic Diversity and Heterogenous Origins of Pathogenic and Antibiotic-Resistant Escherichia coli in Household Settings Represent a Challenge to Reducing Transmission in Low-Income Settings

**DOI:** 10.1128/mSphere.00704-19

**Published:** 2020-01-15

**Authors:** Maria Camila Montealegre, Alba Talavera Rodríguez, Subarna Roy, Muhammed Iqbal Hossain, Mohammad Aminul Islam, Val F. Lanza, Timothy R. Julian

**Affiliations:** a Eawag, Swiss Federal Institute of Science and Technology, Dübendorf, Switzerland; b Servicio de Microbiología, Hospital Universitario Ramón y Cajal (IRYCIS), Madrid, Spain; c Food Microbiology Laboratory, Laboratory Sciences and Services Division, International Centre for Diarrhoeal Disease Research, Dhaka, Bangladesh; d Paul G. Allen School for Global Animal Health, Washington State University, Pullman, Washington, USA; e Swiss Tropical and Public Health Institute, Basel, Switzerland; f University of Basel, Basel, Switzerland; JMI Laboratories

**Keywords:** *Escherichia coli*, genomic diversity, accessory genes, soils, household settings

## Abstract

Escherichia coli is present in multiple hosts and environmental compartments as a normal inhabitant, temporary or persistent colonizer, and as a pathogen. Transmission of E. coli between hosts and with the environment is considered to occur more often in areas with poor sanitation. We performed whole-genome comparative analyses on 60 E. coli isolates from soils and fecal sources (cattle, chickens, and humans) in households in rural Bangladesh. Isolates from household soils were in multiple branches of the reconstructed phylogeny, intermixed with isolates from fecal sources. Pairwise differences between all strain pairs were large (minimum, 189 single nucleotide polymorphisms [SNPs]), suggesting high diversity and heterogeneous origins of the isolates. The presence of multiple virulence and antibiotic resistance genes is indicative of the risk that E. coli from soil and feces represent for the transmission of variants that pose potential harm to people. Analysis of the accessory genomes of the Bangladeshi E. coli relative to E. coli genomes available in NCBI identified a common pool of accessory genes shared among E. coli isolates in this geographic area. Together, these findings indicate that in rural Bangladesh, a high level of E. coli in soil is likely driven by contributions from multiple and diverse E. coli sources (human and animal) that share an accessory gene pool relatively unique to previously published E. coli genomes. Thus, interventions to reduce environmental pathogen or antimicrobial resistance transmission should adopt integrated One Health approaches that consider heterogeneous origins and high diversity to improve effectiveness and reduce prevalence and transmission.

**IMPORTANCE** Escherichia coli is reported in high levels in household soil in low-income settings. When E. coli reaches a soil environment, different mechanisms, including survival, clonal expansion, and genetic exchange, have the potential to either maintain or generate E. coli variants with capabilities of causing harm to people. In this study, we used whole-genome sequencing to identify that E. coli isolates collected from rural Bangladeshi household soils, including pathogenic and antibiotic-resistant variants, are diverse and likely originated from multiple diverse sources. In addition, we observed specialization of the accessory genome of this Bangladeshi E. coli compared to E. coli genomes available in current sequence databases. Thus, to address the high level of pathogenic and antibiotic-resistant E. coli transmission in low-income settings, interventions should focus on addressing the heterogeneous origins and high diversity.

## Abstract (precis)

Escherichia coli is reported in high levels in household soil in low-income settings. When E. coli reaches a soil environment, different mechanisms, including survival, clonal expansion, and genetic exchange, have the potential to either maintain or generate E. coli variants with capabilities of causing harm to people. In this study, we used whole-genome sequencing to identify that E. coli isolates collected from rural Bangladeshi household soils, including pathogenic and antibiotic-resistant variants, are diverse and likely originated from multiple diverse sources. In addition, we observed specialization of the accessory genome of this Bangladeshi E. coli compared to E. coli genomes available in current sequence databases. Thus, to address the high level of pathogenic and antibiotic-resistant E. coli transmission in low-income settings, interventions should focus on addressing the heterogeneous origins and high diversity.

## INTRODUCTION

Escherichia coli is a commensal bacterium but also a versatile pathogen capable of causing intestinal and extraintestinal infections (1, 2). For instance, multiple E. coli pathotypes are among the most important etiological agents of different human infections, such as enteropathogenic E. coli (EPEC) and Shiga toxin-producing E. coli (STEC) of diarrheal disease and extraintestinal pathogenic E. coli (ExPEC) of urinary tract infections (3, 4). However, E. coli is not restricted to human or animal hosts (5), as evidenced by studies demonstrating that E. coli can transit, survive for long periods, and even grow in diverse environmental compartments, such as soil and water (6, 7).

The diversity of E. coli lifestyles is associated with the plasticity of its genome, which is considered open (8). E. coli survival and transit through multiple hosts and environmental compartments likely shaped the evolution and population structure of the species (8). Currently, only 16% of the genes of an E. coli strain belong to the core genome, while the remaining are considered the accessory genome (9). Despite E. coli’s genome diversity, the core genetic structure of the species is clonal, with clear distinction of different phylogenetic groups (phylogroups): seven are part of E. coli *sensu stricto* (A, B1, B2, C, D, E, and F) and the eighth is known as clade I (10, 11). The prevalence and relative abundance of the phylogroups vary among different hosts, ecological niches, and geographic locations (8, 12, 13, 70, 71). However, little is known about the genomic composition of E. coli isolated from open environments (such as soils) and whether specific genetic determinants contribute to survival, adaptation outside the host, or subsequent transmission (6). For instance, some authors have found unique E. coli fingerprints from soils compared to those from animal fecal sources (14), and others have suggested the naturalization of specific E. coli genotypes to soils (7). Luo et al. reported that the genome sequences of nine strains recovered primarily from environmental sources were phylogenetically distinct from commensal or pathogenic host-associated E. coli (15). In contrast, many settings in low- and middle-income countries (LMICs) are characterized by poor or nonexistent sanitary barriers for both people and animals that lead to fecal—and thus E. coli—contamination of environmental compartments (16–18).

Direct contact and close space sharing among multiple hosts (humans, domestic animals, and livestock) in these settings contribute to increased transmission of strains between hosts and with the environment (19). For example, one study in Bangladesh showed that animal feces contribute to higher loads of E. coli in soil, water, and food (18). Contributions of animals to E. coli in soil households in rural Bangladesh were further supported by evidence of ruminant- and avian-associated microbial source tracking markers (BacR and avian-GFD, respectively) in soils (20), and an adjunct study to the water, sanitation, and hygiene (WASH) Benefits Trial in rural Bangladesh stressed the importance of animal feces containment (domestic animals were found to be the key contributors to enteric pathogens in household environments) to reduce transmission of pathogens (21). Moreover, increased prevalence and transmission of resistant E. coli variants have also been linked to the use of antimicrobials, which are often unregulated in LMICs (22, 23). Understanding the dynamics of pathogen transmission is important for the design of effective WASH and One Health interventions.

The present study used comparative genomics, including phylogenetic reconstruction and pairwise differences analysis, to investigate genetic and population-level relationships between E. coli isolates from feces (cattle, chickens, and humans) and soil in households in rural Bangladesh, an area characterized by high disease transmission. E. coli isolates were further characterized by genes associated with virulence, antibiotic resistance, and plasmid replicons. The accessory genome of Bangladeshi E. coli was further analyzed in a broader context by comparison with representative E. coli genomes available in NCBI.

## RESULTS

### Genomic diversity among soils and fecal E. coli isolates from rural Bangladesh.

E. coli collected from soil and feces of humans and animals (chicken and cattle) in households in rural Bangladesh were analyzed using whole-genome sequencing (WGS). The size of the genome assemblies from the 60 E. coli isolates sequenced ranged from 4.52 to 5.50 Mb with a mean GC content of 50.6% (see Table S1 in the supplemental material). Analysis of the seven housekeeping genes used in the Achtman multilocus sequence type (MLST) scheme indicated a high degree of diversity among the sequenced E. coli isolates (Table 1). We found 39 different sequence types (STs) from which 28 STs were represented by only one isolate, while the other 11 STs were represented by at most three isolates. An additional six novel allele combinations were observed among the remaining six isolates (see Table S2). No ST was particularly prevalent in any of the four sources (human, chicken, and cattle feces and soil) studied. Additionally, among the 14 households from which we sequenced E. coli from three or four sources, we found the same ST shared between two isolates in only three of the households (HH18, HH19, and HH24) (Tables 1 and S2).

**TABLE 1:** Characteristics of the 60 isolates sequenced in this study

Location HH^*a*^	Sample	Source	Phylogroup^*b*^	ST^*c*^	Intestinal marker(s)^*d*^	AMR phenotype^*e*^	Acquired gene(s)^*f*^ or chromosomal mutation(s)^*g*^ associated with AMR	Plasmid replicon(s)
Sreehori Para, Mirzapur	HH03C	Cattle	B1	8369	*astA*			ColpVC
	HH03CH	Chicken	B1	2165	*aatA, astA*	SXT^r^	*aadA5*, *dfrA17*, *sul2*	IncY
	HH03H	Human	B1	180		TET^r^	*tet*(A)	
	HH03S	Soil	B1	392				IncFIA(HI1), IncFIB(AP001918), IncFIC(FII)
Sarishadair, Mirzapur	HH08C	Cattle	B1	223		ATM^i^		IncFIB(AP001918), IncB/O/K/Z
	HH08CH	Chicken	A	226	*aatA*	AMP^r^, CFM^r^, CTX^r^, CRO^r^, CAZ^i^, ATM^r^	*bla*_CTX-M-15_, *qnrS1*, *mdf*(A)	
	HH08H	Human	B1	7130	*astA*			IncFIB(AP001918), IncFII(pRSB107)
Andhora, Mirzapur	HH13C	Cattle	B1	155				IncFIA(AP001918), IncFIB(AP001918)
	HH13CH	Chicken	B1	162		TET^r^, AMP^r^, SXT^r^ NAL^r^, CIP^r^	*tet*(B)*, bla*_TEM-1_, *aadA5*, *aph*(*3′'*)*-Ib*, *aph*(6)*-Id*, *dfrA17*, *sul2*, *gyrA*(S83L), *gyrA*(D87N), *parC*(pS80I)	IncFIB(AP001918), IncFIC(FII), IncFII(pSFO), IncQ1, ColpVC
	HH13H	Human	B1	641	*eltA*, *eltB*	AMP^r^, CFM^r^, SXT^i^, AZM^r^	*bla*DHA-1, *dfrA17*, *sul1*, *qnrB4*, *mph*(A)	IncFII(pSFO)
	HH13S	Soil	B1	2073				IncI1(Alpha), Col(BS512)
Dulla Begum, Bhatgram	HH14C	Cattle	B1					IncFIA(AP001918), IncFIB(AP001918)
	HH14CH	Chicken	B1	1326	*aatA*	TET^r^, SXT^r^	*tet*(A)*, aadA5*, *dfrA17*, *sul2*	IncFIB(AP001918), IncFII(pRSB107), IncY
	HH14H	Human	A		*aatA*			
	HH14S	Soil	B1	1656	*astA*			
Dulla Begum, Bhatgram	HH15C	Cattle	E	3233				IncFIB(AP001918), IncFIC(FII), IncFII(pSFO), IncI1(alpha)
	HH15CH	Chicken	A	752	*eae*, *aatA*	AMP^r^	*aph*(*3′′*)*-Ib*, *aph*(*6*)*-Id*	IncFIB(AP001918), IncFIC(FII), IncFII(pSFO), p0111
	HH15H	Human	B2	1193		NAL^r^, CIP^r^, AZM^r^	*erm*(B)*, gyrA*(S83L), *gyrA*(D87N), *parC*(S80I), *parE*(L416F)	IncFIA(AP001918), IncFIB(AP001918), Col(BS512), Col156
Dulla Begum, Bhatgram	HH16C	Cattle	B1	2522		TET^r^	*tet*(B)	
	HH16CH	Chicken	B1	180				
	HH16H	Human	B1	224		TET^r^, AMP^r^, SXT^r^, NAL^r^, CIP^r^, CHL^i^	*tet*(A), *aadA2*, *cmlA1*, *dfrA12*, *sul3*, *gyrA*(S83L), *gyrA*(D87N), *parC*(S80I), *parE*(S458A)	IncFIB(AP001918), IncFII(pSE11), Col(BS512), ColpVC
	HH16S	Soil	B1	40	*astA*	AMP^r^		IncFIA(AP001918), IncFIB(AP001918), IncFIC(FII), IncFII(pSFO), Col(BS512), Col156
K. Deohata, Gorai	HH17C	Cattle	A	2207	*aatA*, *astA*		*catB4*	IncFIB(AP001918), IncFII(pHN7A8), IncFII(pRSB107)
	HH17CH	Chicken	B1	155	*aatA*	TET^r^, AMP^r^, NAL^i^	*tet*(A)	IncFIB(AP001918), IncFII(pCoo), p0111
	HH17H	Human	A	1823				IncHI1A, IncHI1B(R27), ColpVC
	HH17S	Soil	A	10	*aatA*			Col(BS512)
K. Deohata, Gorai	HH18C	Cattle	A	542	*aatA*, *astA*	NAL^r^	*gyrA*(S83L)	IncFIB(AP001918), IncFII(pSFO)
	HH18CH	Chicken	A	542	*aatA*, *astA*	NAL^r^	*gyrA*(S83L)	IncFIB(AP001918), IncFII(pHN7A8), IncFII(pRSB107)
	HH18H	Human	D	106				Col156, ColpVC
	HH18S	Soil	B1	5730	*astA*			IncN, Col(BS512)
Baimhati, Mirzapur	HH19C	Cattle	B1	224				
	HH19CH	Chicken	B1	2160				IncFIB(AP001918), IncFII(pRSB107)
	HH19H	Human	A	10	*aatA*, *astA*	AMP^r^, NAL^r^, AZM^r^	*bla*_TEM-1_, *mph*(A), *gyrA*(S83L)	IncFII(pSFO), Col(BS512), Col(MG828), ColpVC
	HH19S	Soil	B1	2160				IncFIB(AP001918), IncFII(pRSB107)
Baimhati, Mirzapur	HH20C	Cattle	B1	101	*astA*			IncFIA(AP001918), IncFIB(AP001918), IncFIC(FII), IncFII(pSFO), Col(MG828), Col156
	HH20CH	Chicken	B1	111	*aatA*			IncFIB(AP001918), IncFIC(FII), IncFII(pSFO)
	HH20H	Human	B1	224				
	HH20S	Soil	A	10	*astA*	TET^r^, AMP^R^, CFM^r^, CTX^r^, CRO^r^, CAZ^R^, ATM^r^, SXT^r^, NAL^r^, CIP^r^ AZM^r^, CHL^r^	*catA1*, *tet*(B), *bla*_OXA-1_, *bla*_CTX-M-15_, *aadA5*, *aac*(*6'*)*Ib-cr*, *dfrA17*, *sul1*, *mph*(A), *erm*(B), *gyrA*(S83L), *gyrA*(D87N), *parC*(S80I), *parE*(S458A)	IncFIA(AP001918), IncFIB(AP001918), Col(BS512), ColRNAI
Sinjuri, Bhatgram	HH24C	Cattle	B1	101				IncFIA(AP001918), IncFIB(AP001918), IncFIC(FII), IncFII(pSFO)
	HH24CH	Chicken	B1	40	*eae*, *nleA*, *nleC*	AMP^i^		IncQ1
	HH24H	Human	B1	40	*eae*, *nleA*, *nleC*			
Sinjuri, Bhatgram	HH25C	Cattle	A	6622	*aatA*	TET^r^, SXT^r^	*tet*(A), *dfrA14*, *sul2*, *qnrS1*	IncFIB(AP001918), IncFII, IncX4
	HH25CH	Chicken	B1		*aatA*	TET^r^, AMP^i^, SXT^r^	*tet*(A), *dfrA14*, *sul2*, *qnrS1*	IncFIB(AP001918), IncFII(pSFO), p0111
	HH25H	Human	B1	162	*astA*	TET^r^	*tet*(A)	IncFIB(AP001918), IncI1(alpha), ColpVC
	HH25S	Soil	A		*aatA*, *astA*			IncFII(pSFO), IncI2
Sinjuri, Bhatgram	HH26C	Cattle	F^*h*^/B2					IncFIB(AP001918), IncFIC(FII), IncFII, IncFII(pSFO)
	HH26CH	Chicken	Clade I	5273	*aatA*, *astA*			IncFIB(AP001918)
	HH26H	Human	A	206	*eae*, *perA*, *aatA, nleA*, *nleC*	AMP^r^, CFM^r^, CTX^r^, CRO^r^, ATM^i^	*bla*_CTX-M-15_, *qnrS1*	IncFIA(HI1), IncFIB(AP001918), IncFII(pSFO), IncI2(delta), Col156
	HH26S	Soil	A	4	*aatA*, *astA*	TET^r^	*tet*(A)	Col(BS512), p0111
Sreehori Para, Mirzapur	HH29CH	Chicken	A	752	*eae*, *aatA*, *nleA*	TET^r^	*tet*(A), *aph*(*3′′*)*-Ib*, *aph*(6)*-Id*	IncFIB(AP001918), IncFIC(FII), IncFII(pSFO), Col(BS512), ColpVC, p0111
	HH29H	Human	A	48	*aatA*	TET^r^	*tet*(A)	IncHI2, IncHI2A, IncQ1, p0111
	HH29S	Soil	B1	7130				IncFIB(AP001918), IncFII(pRSB107)
Dulla Begum, Bhatgram	HH34S	Soil	B1	155	*aatA*			IncHI1B(CIT)_1_pNDM-CIT, IncY
Baimhati, Mirzapur	HH36S	Soil	A	1585	*aatA*	TET^r^, AMP^r^, CFM^r^, SXT^r^, NAL^r^, CIP^r^, AZM^r^	*tet*(A), *bla*_DHA-1_, *bla*_TEM-1_, *aadA5*, *aph*(*3′*)*-Ia*, *dfrA1*, *dfrA17*, *sul1*, *sul2*, *qnrB4*, *qnrS1*, *mph*(A)*, gyrA*(S83L), *parC*(S80I)	IncFIB(pB171), IncFII, IncI1(alpha), IncX1, Col(BS512), ColRNAI
Sinjuri, Bhatgram	HH41S	Soil	Clade I			TET^r^, AMP^r^	*tet*(A), *bla*_TEM-1_	IncFII, IncHI1A, IncHI1B(CIT)
Satiachara, Jamurki	HH45S	Soil	D	2914				IncFIB(AP001918), IncI1(alpha), Col(BS512)
Sinjuri, Bhatgram	HH46S	Soil	B1	58		AMP^r^, CTX^r^, CRO^r^, CFM^r^, CAZ^i^, ATM^r^	*bl*a_CTX-M-15_, *qnrS1*	
Kanthalia, Mirzapur	HH49S	Soil	B1	3580				
Kanthalia, Mirzapur	HH50S	Soil	B1	75		TET^r^, AMP^r^	*tet*(B), *bla*_TEM-1_, *qnrS1*, *mph*(A)	IncFIA(HI1), IncFIB(AP001918), IncHI1A, IncHI1B(R27), ColpVC
Kanthalia, Mirzapur	HH51S	Soil	B1	1079	*aatA, astA*			IncY

a Location of the household: village, union.

b Phylogenetic group based on the *in-silico* ClermonTyping.

c Sequence type (ST) based on multilocus sequence typing Achtman scheme.

d EPEC: *eae*, *bfp*, and *perA*; EAEC: *aatA*; EIEC: *ipaH* and *ial*; ETEC*: eltA*, *eltB*, and *lt*; EHEC: *espK*, *espN*, *nleA*, *nlec*, and *nleG*; STEC: *astA*, *aaic*, *stx1a*, *stx1b*, *stx2a*, and *stx2db*.

e Antimicrobial resistance (AMR) phenotype by disk diffusion test for tetracycline (TET), the penicillin ampicillin (AMP), the third-generation cephalosporins cefixime (CFM), cefotaxime (CTX), ceftriaxone (CRO), and ceftazidime (CAZ), the monobactam aztreonam (ATM), the inhibitor of the folate pathway trimethoprim-sulfamethoxazole (SXT), the quinolones nalidixic acid (NAL) and ciprofloxacin (CIP), the macrolide azithromycin (AZM), and the pnenicol chloramphenicol (CHL) (only intermediate or resistance phenotypes are reported).

f Acquired resistance genes with identity and coverage of >90% with the ResFinder database.

g The gene and amino acid change and position are indicated.

h Isolate HH26C is assigned to the Clermont phylogroup B2 based on the results of the *in silico* PCR (− + + −), but the Mash genome distance method classifies this strain as phylogroup F.

10.1128/mSphere.00704-19.1 TABLE S1 Assembly statistics and GenBank accession numbers of the 60 genome sequences included in this study. Download Table S1, XLSX file, 0.1 MB. Copyright © 2020 Montealegre et al. 2020 Montealegre et al. This content is distributed under the terms of the Creative Commons Attribution 4.0 International license.

10.1128/mSphere.00704-19.2 TABLE S2 Achtman 7 Gene MLST of the 60 E. coli isolates included in this study. Download Table S2, XLSX file, 0.1 MB. Copyright © 2020 Montealegre et al. 2020 Montealegre et al. This content is distributed under the terms of the Creative Commons Attribution 4.0 International license.

**FIG 1:**
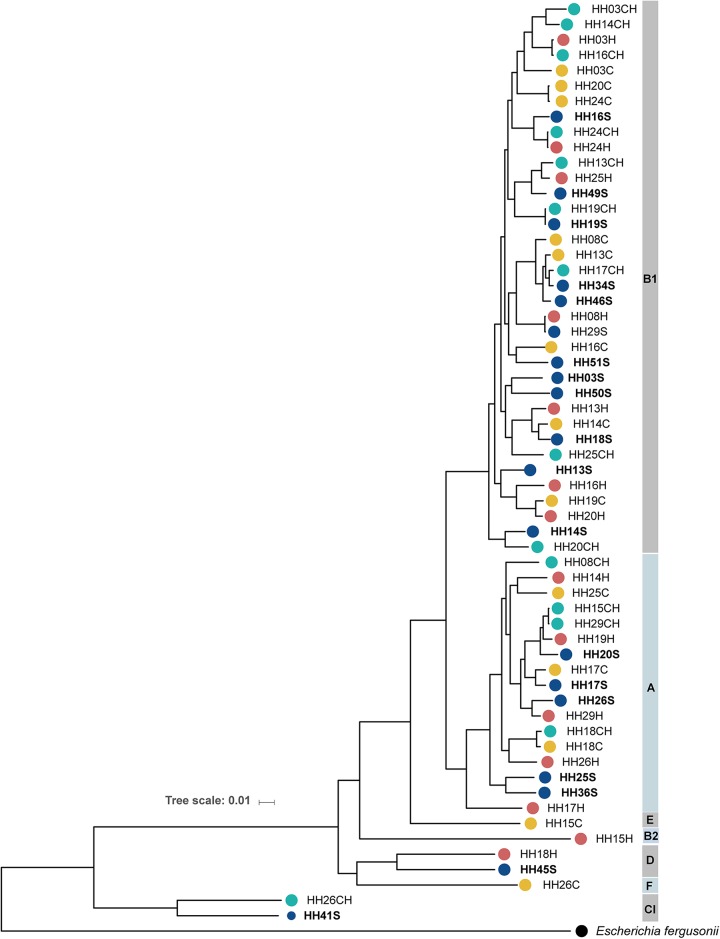
Phylogeny of 60 E. coli isolates collected from soils and fecal sources in rural Bangladesh. The core genome phylogenetic tree, based on SNPs and indels, was constructed by maximum likelihood using IQ tree and visualized using the iTOL online tool. The genome of Escherichia fergusonii was used as the outgroup. The household (HH) where the isolate was collected and the source (“S” for soil, “H” for human fecal, “CH” for chicken fecal, and “C” for cattle fecal) correspond to the isolate name. The source is additionally indicated by colored circles; E. coli phylogroups are indicated on the right.

Comparative genomic analyses of the 60 isolates indicates that the majority of isolates cluster within phylogroups B1 (60%) and A (28.3%); however, rich phylogenetic diversity among the isolates falling in these phylogroups was observed (Fig. 1). A few isolates clustered within phylogroups D (HH18H and HH45S), E (HH15C), F (HH26C), and B2 (HH15H), while two other isolates (HH26CH and HH41S) were closer to a genome from *Escherichia* clade I (Fig. 1 and Table 1). The 19 isolates collected from household soil samples were in multiple branches of the phylogeny, intermixed with isolates from fecal sources (chicken, human, and cattle feces). Only in two instances, E. coli recovered from two different household soils formed a terminal lineage (Fig. 1) (isolates HH25S and HH36S within phylogroup A; HH03S and HH50S within phylogroup B1). The phylogenetic tree also revealed little to no differences among six isolate pairs, two from the same household but different sources (HH19CH:HH19S and HH24CH:HH24H) and the other four from different households located in different villages (Fig. 1 and Table 1) (HH03H:HH16CH, HH08H:HH29S, HH15CH:HH29CH, and HH20C:HH24C). To further study the relationship among these isolates, we performed pairwise comparisons to evaluate the number of single nucleotide polymorphisms (SNPs) in the core genome among each possible isolate pair (see Table S3). Pairwise differences between pairs were generally large, with medians (interquartile ranges) of 21,820 (19,476 to 25,404) SNPs among phylogroup A isolates and 22,374 (21,393 to 23,264) SNPs among phylogroup B1 isolates. The most closely related isolates correspond to the pairs recovered from the same household, HH19CH:HH19S and HH24CH:HH24H, with 189 and 192 SNPs, respectively. One additional closely related pair (203 SNPs) was identified, with isolates from both different households and different sources (HH08H:HH29S). We found no differences in the means of the pairwise differences rankings among isolates from the same household compared to those from different households (Wilcoxon rank-sum test, *P* = 0.11) or from the same source compared to those from different sources (Wilcoxon rank-sum test, *P* = 0.44).

10.1128/mSphere.00704-19.3 TABLE S3 Pairwise comparisons of the number of SNPs in the core genome among each possible isolate pair. Download Table S3, XLSX file, 0.1 MB. Copyright © 2020 Montealegre et al. 2020 Montealegre et al. This content is distributed under the terms of the Creative Commons Attribution 4.0 International license.

### Pathogenic potential of soils and fecal E. coli isolates from rural Bangladesh.

Within the set of 60 isolates, 531 unique virulence factor-related genes were identified with identity and coverage greater than 90% against the 32,312 total (2,681 *Escherichia*) virulence factor-related genes included in the virulence factor database (see Table S4). The number of virulence factor-related genes per isolate was on average (median) 179 (184) and ranged from 117 (HH17H) to 234 (HH26H and HH45S). Among the 531 unique genes, 83 (15.6%) were found in all 60 isolates and 110 (20.7%) were found in at least 54 isolates. More than half (313 genes [58.9%]) were found in less than 6 isolates, with 111 (20.9%) of these detected in only one isolate (Table S4).

10.1128/mSphere.00704-19.4 TABLE S4 Virulence factor-related genes encountered in the 60 E. coli isolates collected from soils and fecal sources in households in rural Bangladesh. Download Table S4, XLSX file, 0.1 MB. Copyright © 2020 Montealegre et al. 2020 Montealegre et al. This content is distributed under the terms of the Creative Commons Attribution 4.0 International license.

The frequently detected virulence factor-related genes are linked not only to virulence but also to environmental adaptation. For example, genes related to acid resistance (*gadX*), cation efflux (*ibeB* and *ibeC*), adhesive curli fimbriae (*csgBAC* and *csgDEFG* operons), and the siderophore enterobactin used for iron acquisition were detected in all isolates. Genes related to the type 1 fimbria operon and flagella were also very common (Table S4).

**FIG 2:**
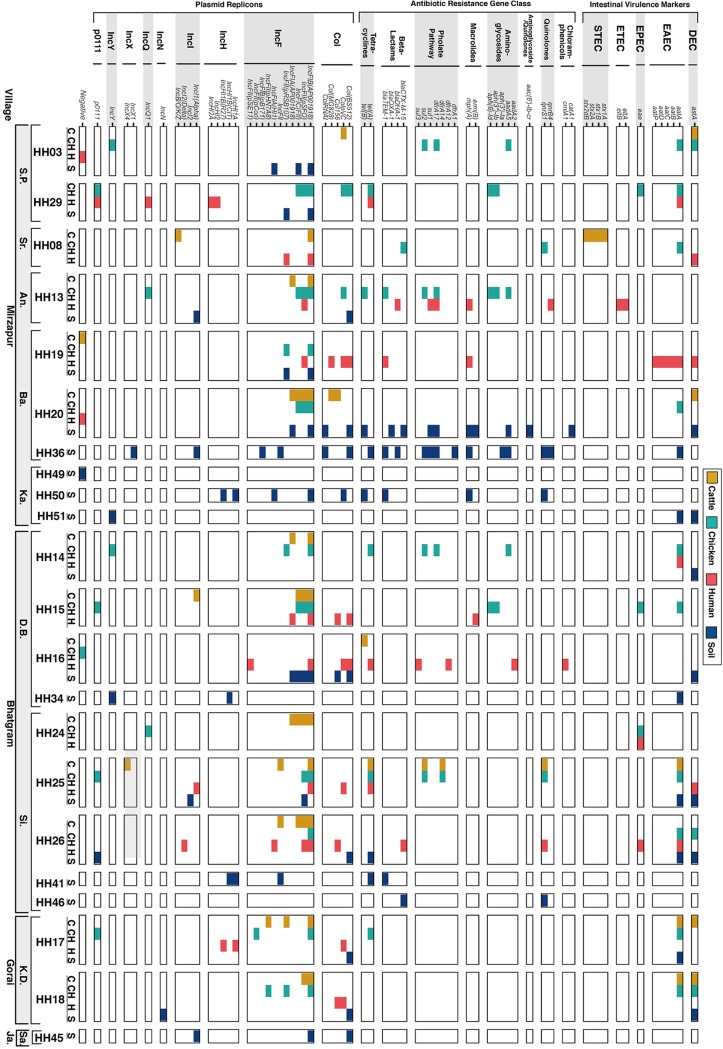
Intestinal virulence markers, antibiotic resistance genes, and plasmid replicon profiles for the 60 E. coli isolates collected from soils and fecal sources in households in rural Bangladesh. Distribution of virulence genes associated with intestinal pathotypes, antibiotic resistance gene determinants, and plasmid replicons with identity and coverage >90% against the Virulence Factor, ResFinder, and PlasmidFinder databases, respectively. The virulence genes are grouped by intestinal pathotype, the antibiotic resistance genes are grouped by antibiotic class, and the plasmid replicons are grouped by plasmid groups (*x* axis). The isolates are grouped by source, household, union, and village (Mirzapur: S.P., Sreehori Para; Sr., Sarishadair; An., Andhora; Ba., Baimhati; Ka., Kanthalia; Bhatgram: D.B., Dulla Begum; Si., Sinjuri; Gorai: K.D., K. Deohata; Jamurki [Ja]: Sa., Satiachara) (*y* axis). The source of isolation is also indicated by the colors.

Identified virulence factor-related genes included multiple genes used as diagnostic targets for intestinal E. coli pathotypes (Fig. 2). The *astA* gene, which encodes a heat-stable enterotoxin and is linked to diarrheagenic E. coli caused by enteroaggregative E. coli (EAEC), EPEC, and noncategorized diarrheagenic E. coli (DEC) (24), was detected in 17 isolates (4 cattle, 3 chicken, 3 human, and 7 soil). The *eae* gene indicating EPEC was detected in five isolates (3 chicken and 2 human). One isolate (HH13H) was a putative enterotoxigenic E. coli (ETEC), as indicated by the presence of the *eltA* and *eltB* genes, common diagnostic markers for heat-labile ETEC, while one cattle isolate (HH08C) was a putative STEC as indicated by *stx1a*, *stx1b*, *stx2a*, and *stx2db* genes. The gene *aatA* (plasmid-associated and used as a diagnostic target for EAEC [25]) was detected in 23 isolates, including three cattle, 10 chicken, four human, and six soil isolates (Fig. 2).

The observed distribution of virulence factor-related genes across the four isolate sources (cattle, chicken, human, and soil) appeared random based on overall prevalence rates for all except four genes (χ^2^ test, df = 3, unadjusted α = 0.05). Specifically, the adhesin *tia* gene appeared in eight cattle, two chicken, and two soil isolates but in no human isolates (χ^2^ =15.1, *P* = 0.002), and the adhesin-related *cah* gene appeared in seven chicken and six soil isolates but only one human and no cattle isolates (χ^2^ = 9.4, *P* = 0.02). Similarly, *leoA*, a gene linked to secretion of the heat-labile enterotoxin (26), was only present in four cattle isolates (χ^2^ = 14.5, *P* = 0.002); ECP_2814, encoding a hypothetical protein, only appeared in four human isolates and two cattle isolates (χ^2^ = 8.5, *P* = 0.036).

### Antibiotic resistance gene profiles and association with phenotypic resistance.

Among the 60 isolates sequenced, 23 harbored at least one antibiotic resistance gene determinant [excluding *mdf*(A), found in all isolates] with identity and coverage greater than 90% against the ResFinder database (Table 1; Fig. 2) (27). Two soil isolates, HH20S and HH36S, harbored the most resistance genes, with 10 and 12 different genes, respectively. Resistance to tetracycline was reportedly predominant in the sampling area (28) and within the subset of isolates selected for this study (16/60 [26.7%]) (Table 1). Not surprisingly, the most prevalent resistance mechanism encountered was the efflux-mediated resistance to tetracycline encoded by *tet*(A) (*n* = 11) and/or *tet*(B) genes (*n* = 4) (Fig. 2). Resistance to ampicillin was also present in these isolates (23.3%), while beta-lactamase-encoding genes were observed in only 10 isolates (Table 1). Resistance to the third-generation cephalosporins cefixime, cefotaxime, and ceftriaxone was observed in four isolates (HH08CH, HH20S, HH26H, and HH46S), explained by the presence of the extended-spectrum beta-lactamase-encoding gene *bla*_CTX-M-15_ (Table 1). Reduced susceptibility to ceftazidime, as reported for CTX-M-15 (29), was observed in these four isolates; however, only isolate HH20S, carrying also *bla*_OXA-1_, was classified as resistant. Resistance to cefixime alone (also a third-generation cephalosporin) was observed in isolate HH13H, harboring *bla*_DHA-1_. The *sul* and *dfrA* genes, associated with class 1 integrons (30) and encoding a dihydropteroate synthase and a dihydrofolate reductase, respectively, were coharbored by nine of the 60 isolates, with intermediate or resistant phenotypes to trimethoprim-sulfamethoxazole (Table 1). Genes associated with resistance to aminoglycosides (*aadA* and *aph* variants) were observed in eight isolates, often from chicken origin (Fig. 2). Indeed, the genes *aph*(*3′′*)*-Ib* and a*ph*(*6*)*-Id* appeared to not be randomly distributed across the four sources, as they were only detected in chicken but not in any of the other sources (χ^2^ = 9.9, *P* = 0.002). The plasmid-mediated quinolone resistance (PMQR) genes *QnrS1* and *QnrB4* were detected in eight E. coli; however, no clinical resistance to ciprofloxacin, based on CLSI breakpoints, was observed in these isolates, except for one soil isolate that coharbored both genes. *QnrS1* and *QnrB4* are known to provide a low level of resistance, while mutations in the genes encoding DNA gyrase and topoisomerase IV are associated with observable resistance to ciprofloxacin and/or nalidixic acid (31), as in the case of seven E. coli isolates of this study (Table 1). Resistance to azithromycin (macrolide), detected only in E. coli from human and soil origin, was observed in the five isolates where the macrolide-associated gene(s) *mph*(A) and/or *ermB* was detected (Table 1).

### Prevalence of plasmid replicons among soils and fecal E. coli isolates from rural Bangladesh.

By using an identity and coverage threshold greater than 90% against the PlasmidFinder database, the numbers of plasmid replicons detected ranged from 1 to 7 among 49 isolates (81.7%), while the other 11 isolates had no hits above the predefined threshold (Table 1 and Fig. 2). Thirty-one plasmid replicons associated with large and small plasmids were identified (Fig. 2). The most prevalent replicons were IncFIB(AP001918) and IncFII(pSFO), detected from the four sources in 32 (53.3%) and 15 (25.0%) isolates, respectively. Nine other IncF replicons were detected with variable presence across the sources (Fig. 2). Among the replicons associated with small plasmids, Col(BS512) was the most prevalent, present in 12 (20.0%) isolates with a distribution across the sources that appeared not random, as it was detected in eight soil, three human, and one chicken isolate but not cattle isolates (χ^2^ = 8.4, *P* = 0.038).

### Phylogenetic distance and accessory genomes analyses of soil and fecal E. coli isolates from rural Bangladesh against representative and nearest E. coli genomes available in NCBI.

**FIG 3:**
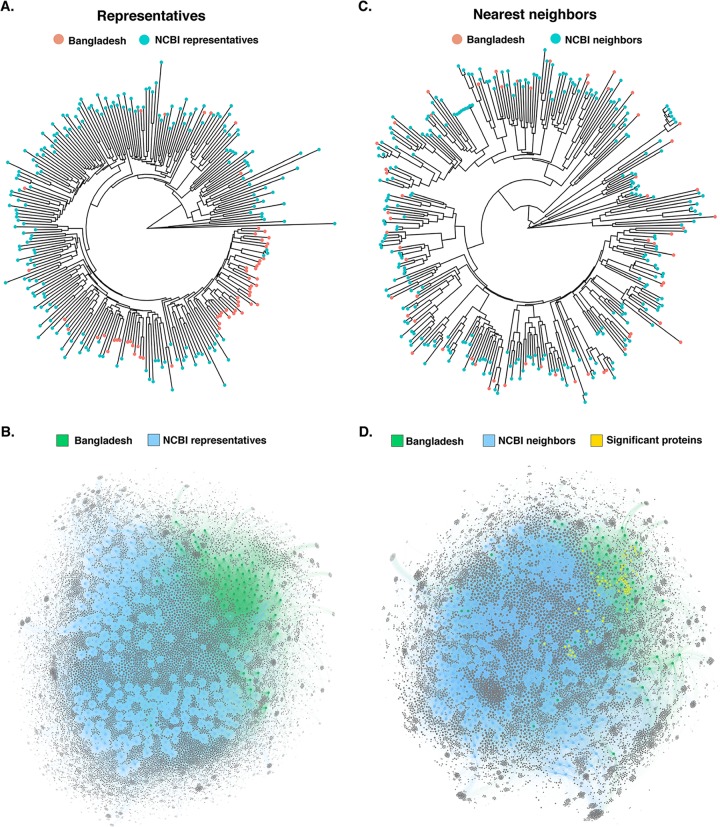
Phylogenetic distance and accessory genome analyses of soil and fecal E. coli isolates from rural Bangladesh against representative and nearest E. coli genomes available in NCBI. Mash distance hierarchical dendrogram of the 60 Bangladeshi E. coli isolates against 199 representative (A) and 265 nearest-neighbor (C) E. coli genomes available in NCBI (see Table S5 in the supplemental material for the list of the genomes used for comparison). Accessory-genome bipartite network generated by AcCNET with the 199 representative (B) and 265 nearest-neighbor (D) accessory genomes. Proteins with a *P* value of <0.001 and frequency in Bangladesh data set of >50% are represented.

We used Mash distance estimation (32) to study the phylogenetic distance of the 60 Bangladeshi soil and fecal E. coli against 199 representative E. coli genomes (Table S5A). The hierarchical dendrogram revealed that isolates of this Bangladeshi collection have, in general, greater sequence similarity among each other than with representatives of the E. coli phylogeny (Fig. 3A). For instance, 23 of the 36 phylogroup B1 Bangladeshi isolates clustered together in the Mash-based dendrogram with only two other genomes (isolated from feces of dogs, ASM332284 and ASM332186) forming part of this cluster. Similarly, 13 of the 17 phylogroup A Bangladeshi isolates formed a cluster, indicating greater similarity among these genomes. As expected, due to the low prevalence of other phylogroups among this Bangladeshi isolate collection, isolates from phylogroups besides A and B1 were scattered among the other genomes (Fig. 3A and S1). The network analysis using the AcCNET (Accessory Genome Constellation Network) application (33) also revealed that the accessory genomes of the Bangladeshi collection have higher similarity among each other than with the accessory genomes of the representative E. coli genomes (Fig. 3B).

10.1128/mSphere.00704-19.5 TABLE S5 List of representative and nearest E. coli genomes used for Mash distance estimation and AcCNET analyses. Download Table S5, XLSX file, 0.1 MB. Copyright © 2020 Montealegre et al. 2020 Montealegre et al. This content is distributed under the terms of the Creative Commons Attribution 4.0 International license.

10.1128/mSphere.00704-19.7 FIG S1 Detailed Mash distance hierarchical dendrogram of the 60 Bangladeshi E. coli isolates against 199 representative E. coli genomes available in NCBI. Download FIG S1, PDF file, 1.8 MB. Copyright © 2020 Montealegre et al. 2020 Montealegre et al. This content is distributed under the terms of the Creative Commons Attribution 4.0 International license.

**FIG 4:**
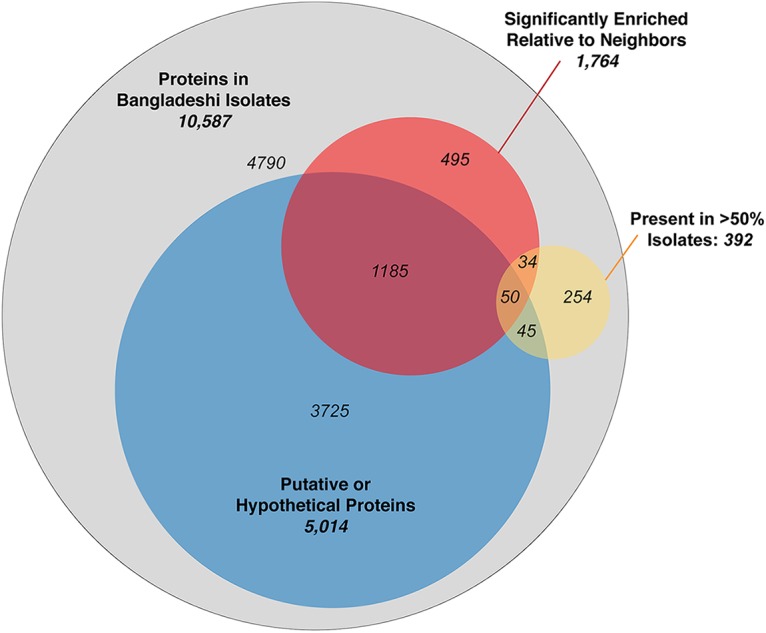
Venn diagram indicating the distribution of the accessory genome proteins found in Bangladeshi E. coli isolates. Proteins coded by the accessory genomes of the Bangladeshi isolates include proteins that are putative or hypothetical with unknown function (47.4%), statistically significantly enriched relative to their nearest neighbors in the NCBI database (16.7%), and/or present in at least half of the Bangladeshi isolates (3.7%).

To identify the genomic characteristics unique to the Bangladeshi isolates, the Mash phylogenetic distance and the frequencies of the protein-coding genes observed within the respective accessory genomes were quantitatively compared to those of the 265 nearest E. coli neighbors (Table S5B). By using the nearest E. coli neighbors, which represent the 10 most closely related E. coli genomes in NCBI for each of the Bangladeshi E. coli isolates (some Bangladeshi E. coli isolates shared the same nearest neighbors), we then observed uniform distance distribution of the Bangladeshi isolates among the E. coli genomes (Fig. 3C), therefore minimizing bias in the subsequent network analyses. AcCNET identified 10,587 protein-coding genes in the accessory genomes of the 60 Bangladeshi isolates and compared the presence/absence frequency to that of 265 nearest E. coli neighbors (Fig. 3D). Of these, 1,764 (16.7%) were statistically significantly enriched in the Bangladeshi E. coli isolates relative to that in genomes of the nearest neighbors (hypergeometric test, Bonferroni adjusted *P* < 0.05) (Fig. 3D and 4). Notably, the accessory genome contained a large proportion of putative or hypothetical proteins with unknown function (5,014 [47.3%]). The proportion of putative or hypothetical proteins was statistically significantly higher (z = −20.9, *P* < 0.001) among the protein-coding genes enriched in the Bangladeshi isolates (1,235/1,764 [70.0%]) than the protein-coding genes shared between the Bangladeshi isolates and the nearest neighbors (3,779/8,823 [42.8%]) (Fig. 4).

The accessory genome analysis identified 84 (0.8%) protein-coding genes that were both statistically significantly enriched and present in at least half of the 60 Bangladeshi isolates (Fig. 4; Table S6). The 84 enriched proteins included putative or hypothetical proteins with unknown function (54 [64%]), proteins coding for domains of unknown function (4 [5%]), or that were otherwise poorly defined (2 [2%]). Among the rest, nine (10%) were related to metabolism (formate dehydrogenase, 6-phospho-alpha-glucosidase, arylsulfatase, fatty acyl-CoA synthetase, peptide chain release factor 2, and carbonic anhydrase), and eight (10%) were related to environmental response, biofilm formation, and/or virulence (murein endopeptidase from DLP12 prophage, response regulators, diguanylate cyclase, fimbrial protein, adhesin-like autotransporter, and flagellar motor rotation) (Table S6). The remaining proteins enriched in the Bangladeshi isolates relative to the nearest neighbors included four (5%) related to insertion sequences IS*1*, IS*2*, and IS*3*; three (3.5%) related to toxin/antitoxin systems for plasmid maintenance, one related to DNA-binding transcriptional regulator, and one related to DNA base-flipping. Notably, 13 of the proteins were not found in any of the 265 nearest neighbors, including DNA base-flipping and formate dehydrogenase H proteins present in all 60 Bangladeshi isolates, and two toxin/antitoxin proteins present in 58 (97%) and 33 (55%) of the Bangladeshi isolates (Table S6).

10.1128/mSphere.00704-19.6 TABLE S6 List accessory genome proteins statistically significantly enriched in Bangladeshi E. coli. Download Table S6, XLSX file, 0.1 MB. Copyright © 2020 Montealegre et al. 2020 Montealegre et al. This content is distributed under the terms of the Creative Commons Attribution 4.0 International license.

## DISCUSSION

We assessed the genomic diversity of E. coli from household soils in a rural Bangladeshi community using WGS and performed comparative analyses with E. coli isolated from feces of potential contributors (human and animal) to shed light on probable sources and transmission patterns. Our findings are indicative of a rich phylogenetic diversity among the E. coli isolates circulating in this rural community with the E. coli isolates recovered from front yard households located in multiple branches of the phylogeny intermixed with isolates from fecal sources (Fig. 1). The high diversity observed among these Bangladeshi E. coli isolates is in line with recent studies in other rural or semirural communities in LMICs (23, 34). For instance, Richter et al. found high interindividual diversity among gastrointestinal E. coli isolates in Tanzanian children and high intraindividual temporal diversity in samples from the same child during a 6-month period (34).

The placement of soil E. coli in terminal lineages of the phylogeny with fecal E. coli suggests the fluidity and lack of phylogenetic structure based on source. Humans and animals are suggested as likely contributors to the E. coli population in soils (28, 35), but clonality or an estimate of the time of diversification between E. coli in soil and E. coli from the input source has not yet been established. Mutation rates are routinely used to establish time of diversification (36, 37). For example, by using estimated mutation rates reported for two different E. coli clones based on the number of differences and approximate time of divergence (2.3 × 10^−7^ to 6.9 × 10^−7^ per site per year) (36, 38), one would predict that 1 to 3 SNPs would arise in 1 year for an average genome size of 4.9 Mbp (the average genome size for the E. coli analyzed in this study). This value is far below the minimum number of SNPs (189 SNPs) observed between the most closely related isolates of this study. Therefore, we found no direct evidence to suggest recent clonal transmission from humans or animals to soils or vice versa. Similarly, other studies have failed to detect recent transmission events between human and animals (domestic and livestock) (13, 23), even when analyzing strains with the same phenotypic resistance (23). In contrast, strain pairs of the 2011 E. coli O104:H4 outbreaks in Germany and France differed by a maximum of 6 and 19 SNPs, respectively (39). However, mutation rate estimates hold several uncertainties. For example, laboratory conditions may not resemble generation times in nature (40, 41) or disregard factors such as differential mutation rates among strains, selection, recombination, and mutational bias (41, 42). Furthermore, little is known on how environmental factors, ecological niches, or different host species affect the rates of accumulation of diversity (39, 43). For instance, differences in diversity were reported even among two different but linked E. coli O104:H4 outbreaks (39). In addition, multiple genome sequences per source must be necessary to understand the origin and patterns of transmission and diversification (39, 40) in a scenario like the one described in this study.

At the core genome level, the Bangladeshi E. coli isolates do not represent a unique population relative to the nearest E. coli neighbors available in the database. Interestingly, when we interrogated their accessory genomes against the nearest E. coli genomes, we observed that approximately one of every six protein-coding genes in the genomes of the Bangladeshi isolates was statistically significantly enriched relative to the nearest E. coli neighbors (Fig. 3 and 4). Protein-coding genes enriched in the Bangladeshi isolates were significantly more likely to code for putative or hypothetical proteins of unknown function than genes shared between the isolates and their nearest neighbors. The clustering of Bangladeshi isolates and the high rate of putative proteins indicate a potentially large pool of unknown biological functions unique to this E. coli community. Known functions enriched in this community included those linked to DNA methylation and repair as well as metabolic processes, suggesting potential adaptive strategies unique to this environment. Together, these findings indicate the cohesiveness of the accessory genomes of this Bangladeshi E. coli population relative to E. coli sequences in the NCBI database while suggesting that the diversity of the accessory genome of even an organism as well studied as E. coli is not completely explored. These findings affirm that certain geographic regions (i.e., Asia) are underrepresented in current sequence databases describing E. coli and associated biological functions, as similarly suggested with recent studies of metagenome-assembled genomes (MAGs) from the gut microbiome (44, 45). In addition, the observed specialization of the accessory genome over the core genome seems to indicate the existence of evolutionary pressure for adaptation to this environment. These results highlight the well-known but perhaps underestimated genomic plasticity of E. coli. Furthermore, the enrichment and sharedness of certain accessory genes suggest an intensive horizontal gene transfer activity among this Bangladeshi E. coli collection.

Bangladeshi E. coli isolates carried multiple virulence factor-related genes, including diagnostic markers for intestinal E. coli pathotypes. For instance, the genes *aatA* and *astA*, associated with EAEC (a pathotype identified as a common cause of child diarrhea in developing and industrialized countries [46]), were prevalent and found in E. coli from the four sources, including soil (Fig. 2). Notably, nine phylogenetically diverse E. coli (median, 37,617 SNPs), including three soil isolates (HH25S, HH26S, and HH51S), coharbored *aatA* and *astA* (the simultaneous presence of *aatA* and *astA* has been associated with prolonged diarrhea [47]), highlighting the diversity of pathogenic E. coli circulating in these rural Bangladeshi communities. The presence of *astA* in the absence of additional pathogenic markers, as observed in eight E. coli isolates, lacks the discriminatory power to assign these strains within any of the intestinal pathotypes, as *astA* has been associated with multiple intestinal pathotypes (48–50) and is also prevalent in extraintestinal (51), commensal (50), and environmental isolates (52). However, the presence of *astA*, even in the absence of other markers, has been associated with important diarrhea outbreaks (53); therefore, its presence in E. coli from soils should not be overlooked. Other intestinal pathotypes (EPEC, ETEC, and EHEC) were not detected in E. coli isolated from soils but were found in isolates from human, chicken, and cattle feces. Overall, these findings are indicative of the potential that E. coli isolated from soils has to cause disease in people. Furthermore, the presence of one or more antibiotic resistance genes in soil isolates (i.e., 12 genes in isolate HH36S) is indicative of the risk that soil E. coli may represent for the transmission of resistant determinants. Indeed, at least one E. coli isolate from soil carried a gene associated with each of the antibiotic resistance gene classes encountered (Fig. 2). Plasmid replicons were also present among this Bangladeshi E. coli collection (81.7%), with no significant difference in the numbers of replicons observed across the sources. Salinas et al. showed that human and domestic animals shared plasmid replicons; however, diversity in the sequences indicated that the plasmids compared were not identical (23). Similarly, soil, human, and animal E. coli of this study share plasmid replicons [i.e., IncFIB(AP001918) and ColpVC]; however long-read sequencing would be necessary to establish if the same plasmid is circulating across reservoirs. In contrast, other replicons were absent from one or more of the studied sources [i.e., Col(BS512)], which suggests that ecological factors and/or the genetic makeup of the E. coli circulating within specific hosts could affect the distribution of certain plasmids replicons. However, the apparent enrichment by sample source may be random for at least some—if not all—of the four virulence genes, two antibiotic resistance genes, and one plasmid replicon as a consequence of the large data set, liberal statistical significance cutoff, and purposive sampling. Nevertheless, the genes are discussed here to inform potential further investigations of source-specific adaptation of E. coli.

The findings have important implications for interventions intending to address the high loads of E. coli contamination in low-income settings. First, the pathogenicity potential and acquired antibiotic resistance of environmental strains reaffirm the need for interventions that effectively reduce E. coli across different environmental reservoirs. This represents a major challenge, as multiple previous studies showed no significant impact of sanitation (16), household-level water, sanitation, and hygiene infrastructure (17, 28) or an integrated water, sanitation, and hygiene intervention (54) on E. coli concentrations in soils in and around households. Second, the lack of core phylogenetic signal based on source and apparent fluidity of E. coli strains across human, animal, and environmental reservoirs reaffirms the need for integrated interventions that address both human and animal fecal sources (One Health approaches) (55). Infection control interventions targeting only people, such as vaccination or traditional drinking water treatment, household sanitation, and hand hygiene services, may be insufficient to meaningfully impact zoonotic reservoirs. Overall, new approaches, potentially including those described as transformative (56, 57), are needed to address the high loads of E. coli contamination in low-income settings that seek to address the heterogeneous origins and high diversity in order to reduce prevalence and transmission.

## MATERIALS AND METHODS

### Bacterial isolates and antibiotic susceptibility testing.

A subset of 60 isolates, part of a 175-isolate collection that was previously recovered in a study conducted in households in rural villages of Mirzapur, Bhatgram, Gorai, and Jamurki in Tangail district of Bangladesh (28), were selected for this study (Table 1). These isolates were phenotypically identified as E. coli using the API-20E (bioMérieux, Marcy-l’Étoile, France). The isolates selected were recovered from 22 households and up to four different sources and included E. coli isolated from front yard soils (*n* = 19) and fecal samples from human (*n* = 14), chicken (*n* = 14), and cattle (*n* = 13) (Table 1). For 14 households, the E. coli isolates included (*n* = 52) were isolated from three or four of the four sources studied, while the remaining isolates (*n* = 8) correspond to E. coli isolated from eight different household soils (Table 1). The nomenclature indicates the household (HH) from which the isolate was collected, followed by the source: “S” for soil, “H” for human fecal, “CH” for chicken fecal, and “C” for cattle fecal (i.e., HH03C is an E. coli isolate from cattle feces in household 03). Disk diffusion against 16 different antibiotic disks was previously performed (28). In addition, susceptibility against azithromycin (AZM) (Oxoid, Basingstoke, UK) was evaluated for selected isolates and interpreted using the Clinical and Laboratory Standards Institute (CLSI) guidelines and interpretation standards (58).

### DNA extraction and whole-genome sequencing.

DNA was extracted from an overnight culture using the DNeasy Blood & Tissue kit (Qiagen, Hilden, Germany) according to the instructions of the manufacturer. Purity and concentration of the DNA were evaluated with a NanoDrop 2000 spectrophotometer (Thermo Scientific) and a Qubit 2.0 fluorometer (Life Technologies), respectively. Libraries were prepared with the Nextera XT kit, and paired-end sequenced was performed using the Illumina HiSeq platform (2 × 150 bp) (Illumina, San Diego, CA, USA).

### Bioinformatic analyses.

Quality of the reads was assessed using FastQC version 0.11.4, available at https://www.bioinformatics.babraham.ac.uk/projects/fastqc/. Reads were *de novo* assembled using SPAdes genome assembler version 3.11.1 (59). Quality of the genome assemblies was evaluated with Quast (60). Genome annotation was performed using Prokka 1.12 (61). The sequence types of the isolates were determined by analyzing the seven housekeeping genes of the multilocus sequence typing (MLST) Achtman scheme using MLST v. 2.16.1 (https://github.com/tseemann/mlst) (62, 63). Phylogenetic group assignation was performed using an *in silico* PCR-based method (64) available at http://clermontyping.iame-research.center/. Identification of core genome SNPs and indels was performed with Snippy 4.0 (65). A core genome phylogenetic tree based on aligned SNPs and indels was constructed by maximum likelihood using IQ tree available at http://www.iqtree.org/. The tree was visualized using ITOL version 4.3.3, available at https://itol.embl.de (66). The presence of antimicrobial resistance genes, putative virulence factors, and plasmid replicons was studied using ABRicate with the ResFinder database (27), Virulence Factor database (VFDB) (67), and PlasmidFinder database (68) (query date, March, 2019; cutoffs, identity and coverage of >90%) (69). Chromosomal mutations associated with antimicrobial resistance were identified using PointFinder available at https://cge.cbs.dtu.dk/services/ResFinder/ (27).

### Phylogenetic distance and analysis of the accessory genomes.

Phylogenetic distance was estimated using Mash (32), while the Accessory Genome Constellation Network (AcCNET) (33) was used to extract the accessory genome proteomes and generate a bipartite network that links the genomes that share a protein. Visualization of the network was performed using Gephi (https://gephi.org/). Analyses were performed using the 60 Bangladeshi soil and fecal E. coli isolates against 199 nonredundant E. coli genomes representative of each branch of the E. coli phylogeny and against 265 nonredundant nearest E. coli genomes, which represent the 10 most closely related E. coli strains for each of the Bangladeshi E. coli isolates, which may be shared among some Bangladeshi E. coli isolates.

### Statistical analyses.

Statistical analyses were performed using R, version 1.2.1335. Pairwise differences in the means of the ranks of the number of SNPs among isolates from the same household or different households and from the same source or different sources were evaluated using the Wilcoxon rank-sum test. To investigate enrichment of virulence factors, antibiotic resistance genes, and plasmid replicons by source, a chi-squared test was used and a *P* value of <0.05 was considered significant.

### Accession number(s).

This whole-genome shotgun project has been deposited at DDBJ/ENA/GenBank under the accession numbers VNWZ00000000 to VNZG00000000 presented in Table S1 in the supplemental material.
