# Supplementary material for: High Genomic Diversity and Heterogenous Origins of Pathogenic and Antibiotic-Resistant Escherichia coli in Household Settings Represent a Challenge to Reducing Transmission in Low-Income Settings
Source: mSphere. 2020 Jan 15;5(1):e00704-19. doi: 10.1128/mSphere.00704-19 (PMC6968650; doi:10.1128/mSphere.00704-19)
Supplement: FIG S1 [file mSphere.00704-19-sf001.pdf]

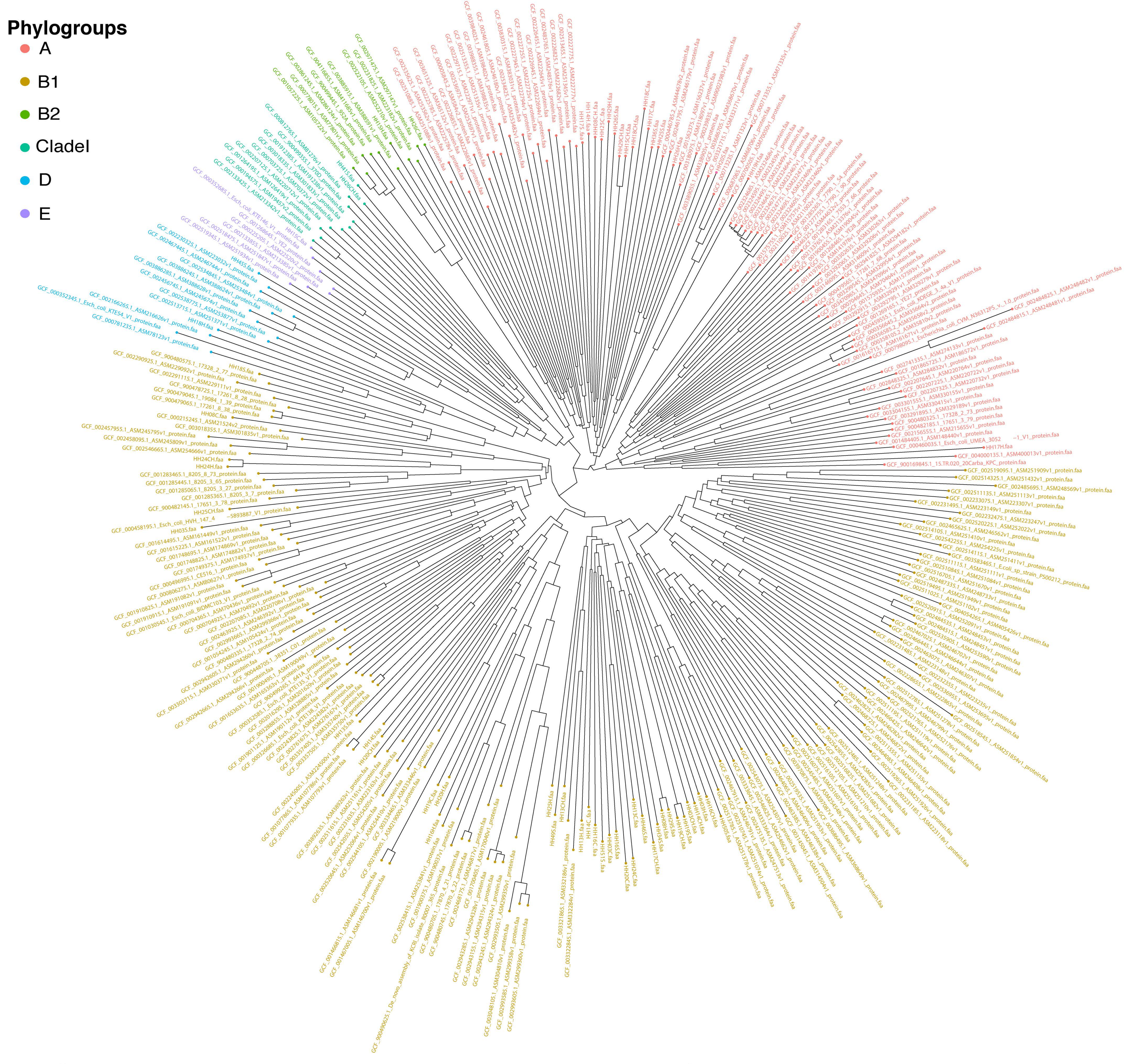

Fig. S1. Detailed Mash distance hierarchical dendrogram of the 60 Bangladeshi *E. coli* isolates
